# Genome sequence analysis provides evidence that a boreal crustacean colonised Svalbard well before the ongoing Atlantification of the Arctic

**DOI:** 10.1038/s41437-025-00793-7

**Published:** 2025-08-23

**Authors:** Hedvig Kriszta Csapó, Jan Marcin Weslawski, Nova Mieszkowska, Ida Dahl-Hansen, Michael Gantsevich, Michal Grabowski, Nicolas Bierne

**Affiliations:** 1 Institute of Oceanology Polish Academy of Sciences, Sopot, Poland; 2 Department of Invertebrate Zoology & Hydrobiology, University of Lodz, Lodz, Poland; 3 The Marine Biological Association of the UK, Plymouth, UK; 4 University of Liverpool, Liverpool, UK; 5 Akvaplan-niva, Fram High North Research Centre, Tromsø, Norway; 6 Moscow State University, Moscow, Russia; 7 Institute of Evolutionary Science of Montpellier, Montpellier, France

**Keywords:** Population genetics, Phylogenetics

## Abstract

The study of present-day species distributions often raises questions about historical demography. A particularly interesting phenomenon to put in historical context is contemporary human-induced atlantification and its role in reshaping Arctic ecosystems. Despite this, the colonisation history of the Arctic remains generally understudied. In this study, we investigated the demographic history of the northern acorn barnacle, *Semibalanus balanoides*, a typically boreal species on the Svalbard Archipelago. Our focus was to determine the source and timing of its colonisation of this Arctic archipelago. Using low-coverage whole-genome sequence data, we evaluated two competing hypotheses: whether *S. balanoides* populations colonised Svalbard through ancient natural processes before the Anthropocene, or if their appearance is more recent, either natural or a consequence of growing anthropogenic influences, such as increased connectivity and global warming. Our results suggest that this boreal species expanded into the Arctic during the later phase of the Holocene Thermal Optimum, well before human-induced climate change.

## Introduction

The Atlantification of the Arctic is a current hot topic in polar research. It is a complex phenomenon mainly induced by an increased inflow of warm Atlantic water to the Arctic (Polyakov et al. 2023). Upon general consensus, Atlantification is used as a blanket term to describe physical, chemical and biological changes of Arctic ecosystems, which make those more similar to boreal (Atlantic) zones. This includes boreal organisms that may react to changing climate regimes by shifting their geographic distributions towards higher latitudes. This effect coupled with the physical restructuring of Arctic environments may promote boreal organisms to successfully establish populations in the High Arctic (Csapó et al. 2021; Ingvaldsen et al. 2021). Such a northward distribution shift has been described in a variety of marine organisms at several trophic levels (Descamps and Strøm 2021; Feng et al. 2016; Fossheim et al. 2015; Priest et al. 2023). Most of the literature refers to Atlantification as a present-day phenomenon, induced by the climate warming of the Anthropocene, mainly the past 100 years. However, Csapó et al. (2021) pointed out the importance of potential ‘past Atlantification events’ in the context of (re)colonisation of Arctic habitats by boreal organisms.

Nowadays, Arctic marine ecosystems consist of elements of both the Arctic and the boreal fauna and flora (Hardy et al. 2011). Although this structure underlies the notion of ongoing Atlantification processes, it must be remembered that Arctic ecosystems are relatively young in evolutionary terms Therefore, one of the most prominent forces in shaping the evolution of polar ecosystems in the northern hemisphere has been the repeating cycles of glacial and interglacial periods of the Pleistocene. During glacial periods, the majority of today’s Arctic and boreal zones along sea shelves had become uninhabitable for most organisms due to extensive ice-sheet covers (Wares and Cunningham 2001; Hijmans et al. 2005; Waltari and Hickerson 2013). Then, interglacial warming periods allowed for those habitats to be once again recolonised. Jenkins et al. (2018) in their meta-analysis of the phylogeography of 21 coastal species in the northeastern Atlantic detected signals of rapid population expansion postdating the Last Glacial Maximum (LGM). The sources of Arctic immigration vary. Some studies highlight the importance of past trans-Arctic connections with the Pacific Ocean in shaping the present day species composition of the European Arctic. The pacific herring (*Clupea pallasii*) established populations in the Barents and White Seas during the Holocene Thermal Maximum (ca. 10–8 kya) (Laakkonen et al. 2013). Similarly, postglacial pacific immigration to the Norwegian Arctic was noted in the case of the bivalve *Mytilus trossulus* (Väinölä and Strelkov 2011). Another way for marine species to recolonise postglacial Arctic habitats is from refugial areas along North Atlantic coasts (Maggs et al. 2008). Populations of the boreal crustacean, *Gammarus oceanicus* for example underwent major expansion events in the past few 1000 years along the Arctic coasts including Svalbard, Iceland, Greenland and the Hudson Bay (Grabowski et al. 2019; Csapó et al. 2021). The species most probably survived the LGM in refugia on the Faroe Islands (Krebes et al. 2011). Similarly, in the case of the cockle *Cestoderma edule* genetic signal suggested the existence of northern glacial refugia (Krakau et al. 2012). A subsequent colonisation of the Norwegian and Barents Seas resulted in the formation of a structured metapopulation in these areas (Genelt-Yanovskiy et al. 2018). As for the colonisation history of the High Arctic, there are much less available studies. In 2002, the blue mussel *Mytilus edulis* was found to once again establish populations on the Svalbard archipelago, after being absent from this area in the past 1000 years (Berge et al. 2005). Svalbard populations were shown to be genetically the closest of those of the eastern Atlantic, while their introduction was hypothesised to be aided by human activities (ship ballast water, floating plastic debris) (Kotwicki et al. 2021). The story of *Mytilus edulis* can be treated as strong evidence for the modern day atlantification of Svalbard. When it comes to other boreal species currently inhabiting the same High Arctic habitats, their area of origin and the time of colonisation is largely unknown.

**Fig. 1:**
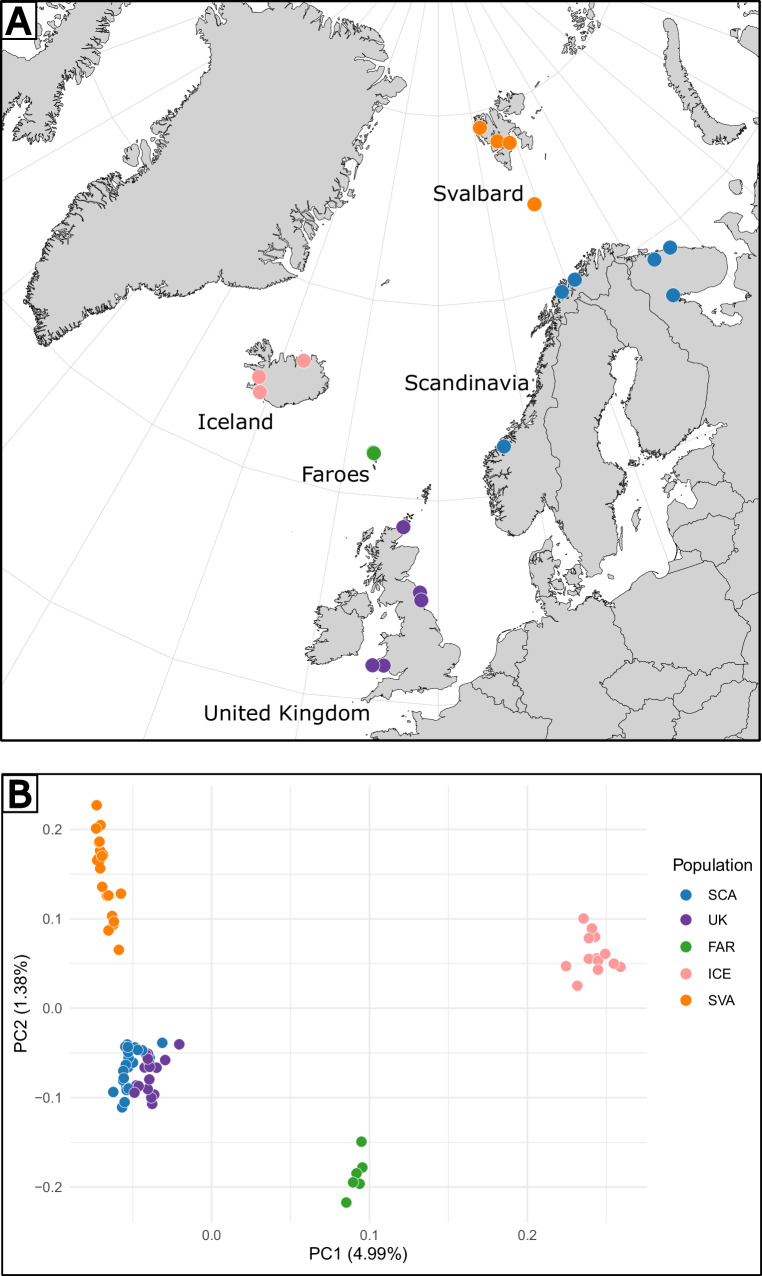
Geographic distribution of samples. **A** Sampling localities for the present study and **B** principal component analysis (PCA) showing the structuring of genetic differences between all samples.

The northern acorn barnacle, *Semibalanus balanoides*, is an intertidal crustacean, with highly dispersive pelagic larval stage and sessile, encrusting adult stages (Anderson 1994). It is a boreal species present in the Arctic, the Atlantic and the Pacific oceans. Its presence was recorded on Svalbard as early as the beginning of the 20th century (Barnes 1957). *S. balanoides* is considered to have a high dispersal potential, in addition to pelagic larvae, the adult stage is also capable of long-distance dispersal by rafting on floating substrata as well as utilising anthropogenic transport via biofouling (Brandler and Carlton 2023; Weslawski and Kotwicki 2018). The North Atlantic biogeography of the species has been studied both with mitochondrial markers (Wares and Cunningham 2001, Nunez et al. 2018, Csapó et al. 2023) and whole genome pool-sequencing data (Nunez et al. 2021). The latter study suggested genome-wide divergence with no recent gene flow between east- and west-Atlantic populations of *S. balanoides*, but lacked samples from the High Arctic region, thus could not shed light on that part of the species history. A recent single-marker biogeographical study by Csapó et al. (2023) investigated the species’ biogeographic history on Svalbard. In general, the Svalbard population was most similar to that of Scandinavia (Fig. 1). However, the genetic resolution of a single marker was not sufficient to infer demographic parameters (colonisation time, gene flow) with precision.

Thus, the aim of the present study is to utilise whole genome sequence data to reveal the demographic history of *S. balanoides* in the European Arctic, with a particular focus on the population of the Svalbard Archipelago. We propose two alternative hypotheses considering the time of Arctic colonisation: (1) Presence of *S. balanoides* populations in Svalbard is the result of recently changing climate conditions favouring the settlement of boreal species in the Arctic. In the case of this scenario, we expect little divergence of the Svalbard population from that of Europe. Divergence times should be not older than 100 or 200 years. Possible genetic differentiation could be explained by a demographic bottleneck during recent colonisation (i.e. founder effect). (2) The divergence of the Svalbard population from its source is older, potentially dated after the end of the LGM. Under such circumstances, we predict Svalbard populations to be significantly divergent from those of other study areas.

## Materials and methods

### Sample collection

We collected 76 individuals of *S. balanoides* from 18 localities across the East-Atlantic/European Arctic (Fig. 1A, Supplementary Table 1). Sampling was carried out according to local legislation, between 2018 and 2022. Permit to collect and export material from Iceland was issued by the Icelandic Institute of Natural History. Individuals were scraped from intertidal rocks with a knife, preserved in 96% ethanol and stored at −20 °C. All specimens used in this study have been deposited in the Department of Hydrobiology and Invertebrate Zoology collection at the University of Lodz in Lodz, Poland.

### DNA extraction and low coverage whole genome sequencing

Individuals were dissected under a stereomicroscope, and a few cirri were taken for DNA extraction. Whole DNA was extracted with the help of A&A Biotechnology Genomic Mini Kit or EurX GeneMATRIX Tissue DNA Purification Kit. DNA concentration of each extract was measured by Nanodrop Spectrometer (Thermo Fisher Scientific Inc.). Individual-based genomic libraries were prepared for each sample with the help of Illumina DNA Prep Kit (Illumina Inc.). Double-strandedness of genomic libraries was assessed with Qubit Fluorometric Quantification (Thermofisher Scientific). Libraries were then pooled based on the molarity of each sample and sequenced across four lanes with Illumina NovaSeq 6000 System (Illumina Inc.), targeting an average 2–3× coverage. Library preparation and sequencing were conducted in BioBank at the Faculty of Biology & Nature Protection, University of Lodz, Poland.

### Data processing and calling genotype likelihoods

Raw reads were demultiplexed with bcl2fastq (Illumina Inc.), quality checked with FastQC (Andrews 2010), and trimmed with the help of trimmomatic 0.39 (Bolger et al. 2014) to keep only high-quality reads (-phred33, SLIDINGWINDOW:4:20, MINLEN:40). Reads that passed all the filters were mapped to the *S. balanoides* reference genome (Sbal_3.1, GCA_014673585.1) with bwa-mem2 (Vasimuddin et al. 2019). All samples had a mapping coverage of ~2.5–4×. In addition, four already published (Nunez et al. 2021) samples were added to our dataset (SRA SRS5300076; SRS5300073; SRS5300071; SRS5300068), representing single individuals from the United Kingdom, Iceland, Norway and the Western Atlantic coast (Rhode Island). These files were downsampled before being processed with the aforementioned pipeline. In result, the final dataset included sequences of 81 *S. balanoides* individuals.

In order to make the best use of the information accessible by low-coverage sequencing, we employed ANGSD to call genotype likelihoods for downstream analyses (Korneliussen et al. 2014). The first run of ANGSD served to find variant sites which pass quality filters in order to call genotype likelihoods (-uniqueOnly 1 -remove_bads 1 -only_proper_pairs 1 -minInd 62 -doCounts 1 -setmaxdepth 810 -skipTriallelic 1 -minMapQ 20 -minQ 20 -GL 1 -doGlf 2 -doMajorMinor 4 -doMaf 1 -nThreads 8). We have filtered out variants which were out of Hardy-Weinberg equilibrium (HWE). In order to do this, we ran ANGSD with the -doHWE flag, then filtered out each site, where *p* values for HWE were smaller than 0.05. Then, the dataset was pruned to account for linkage disequilibrium (LD). First, we took every 10th site to perform LD-pruning to reduce computational time. The programme ngsLD (Fox et al. 2019) was used to calculate LD between SNP-s up to 10 kb distance away, based on genotype likelihoods (--probs --max_kb_dist). Subsequently, LD pruning was performed by ngsLD max 200 kb distance between nodes, and with a minimum weight of 0.5 (--max_dist --min_weight). Lastly, a run of ANGSD was conducted in order to call polymorphic sites (-SNP_pval 1e-6) using the sites already passing the quality filters from the previous runs. Output files of these two runs were subsequently used for downstream analyses. Our carefully filtered final dataset is composed of 100,413 polymorphic LD-pruned variant sites.

### Population structure

The covariance matrix of polymorphic sites including all samples was generated by pcangsd (Meisner and Albrechtsen 2018). It was then used to conduct principal component analysis (PCA) with the help of the eigenfunction in R v. 4.3.2. (R Core team 2023). Individual points on the PCA were coloured by the sampled populations. Two additional PCA plots were prepared. One, including one sample from the Western Atlantic (Rhode Island) (SRA SRS5300068), to understand the genetic proximity of Eastern Atlantic populations to those of the Western Atlantic. As only one sample was available from this region, we did not include it in subsequent, population-level analyses. The second analysis included individuals only from Svalbard and Europe (Scandinavia and British Isles) in order to uncover potential substructure.

Individual ancestry proportions were calculated using a maximum likelihood approach implemented in the programme ngsAdmix (Skotte et al. 2013). We tested for a number of hypothetical populations (K), ranging from 1 to 7. For each K, ngsAdmix was run in 100 replicates. The best likelihood of each group of runs was chosen and compared by CLUMPAK method (Kopelman et al. 2015) in R. The calculated probabilities for each K are provided in Supplementary Table 2.

### Summary statistics: Tajima’s D and nucleotide diversity

Summary statistics for each group identified by the population structure analyses were calculated with ANGSD in order to detect signals of potential bottleneck events. First, we estimated the site allele frequency likelihood (-doSaf 1) for each population. Then, saf files were used to calculate folded single population site frequency spectra (SFS) with realSFS programme, which is part of ANGSD. After that, the realSFS (-saf2theta) and the thetaStat (-do_stat) programmes were used to calculate various summary and neutrality statistics in 50 kb windows along the genome for each population (-win 50,000 -step 10,000). Nucleotide diversity was calculated within each window by dividing the pairwise theta values (tP) by the number of sites (nSites). Raincloud plots in R illustrated the distribution of nucleotide diversity and Tajima’s *D* (Tajima 1989) values along the genome.

### Demographic reconstruction

Two-dimensional folded SFS were estimated for each pair of populations identified by the structure analyses from saf files with realSFS. The Europe (EUR) and Svalbard (SVA) populations were downsampled to 13 individuals to match the number of individuals in the Iceland (ICE) population. The Faroe (FAR) population was omitted from the demographic reconstruction to reduce the number and complexity of tested scenarios. 2D-SFS files were used as input for fastsimcoal27 (Excoffier et al. 2021).

Eight different demographic scenarios were tested with the implemented maximum likelihood method in fastsimcoal (--foldedSFS -m -M). Starting from the simplest scenario (SC1), without gene-flow, SC1-SC6 were designed to test the likelihood of the exchange of genes between the different populations. The following two scenarios then tested for the presence of population growth in the SVA and EUR populations. Exponential population growth following the EUR-SVA divergence was implemented in SC7, while SC8 accounted for population expansion, which predates the aforementioned split. Runs consisted of 50 ECM cycles, 100,000 simulations each (-L 50 -n 100,000). Out of the 50, the first 10 cycles were based on mono- and polymorphic sites, while the remaining cycles optimised the reconstruction likelihood based only on polymorphic sites (-l 10). As a part of the optimisation process, the parameter ANC2 (Supplementary Table 3) was used as a reference after the initial 10 cycles. The inferred rescaling factor was used at the end to adjust the population size and divergence time parameters. Every scenario was run in 100 replicates. The parameters of the replicate with the best likelihood of each scenario were then used as input for likelihood approximation, which was again run in 100 replicates. As a genome-wide mutation rate (μ) is not available for *S. balanoides*, we have followed the approach of Nunez et al. (2020) and used estimates of mutation rates of four arthropod species. We have defined a ‘MUTRATE’ parameter in our analyses and inferred the most likely value between μ = 2.8–3.6 × 10^−9^.

## Results

### Population structure

Principal components explained the following percentages of variance in the data: PC1: 4.99%; PC2: 1.38%; PC3: 1.25%; PC4: 1.09%; PC5: 0.99%. The PCA generally displayed a strong geographic influence on the genetic variance. All individuals from both the Faroe Islands and Iceland clustered in two separate groups, which do not include specimens from any other regions (FAR and ICE). Individuals from Europe (British Isles and the Scandinavian Peninsula) form the third group (EUR). Lastly, a fourth group is formed by individuals from Spitsbergen together with the ones from Bjornoya Island (SVA). Samples from SVA form an elongated cloud along the PC2 axis and appear to be genetically heterogeneous. The one sample from the Western Atlantic coast did not group together with any of the populations. This individual showed genetic proximity to ICE and was positioned between ICE and EUR on the PCA (Supplementary Fig. 1). The higher resolution PCA including individuals only from EUR and SVA revealed two potentially district groups within the SVA population. The EUR population formed one cloud with individuals from the Norwegian (NOR) and Barents Sea (BAR) displaying large within-group genetic variation (Supplementary Fig. 2).

**Fig. 2:**
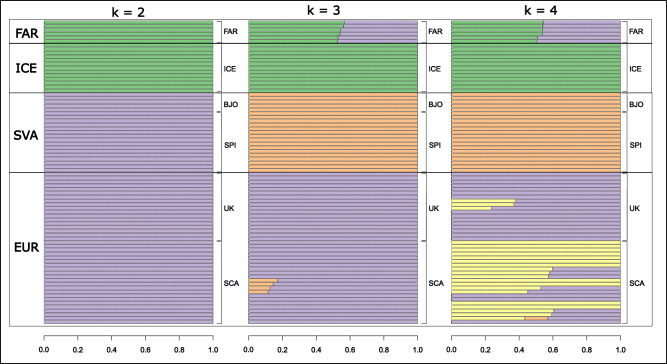
Admixture plots showing individual ancestry proportions of each studied individual for the number of demographic units *k* = 2–4. Subregions: Faroe Island (FAR), Iceland (ICE), Bjornoya Island (BJO), Spitsbergen (SPI), United Kingdom (UK), Scandinavia (SCA).

The admixture analysis provides a similar picture. The most likely scenario was the one with K = 3 (Supplementary Table 2). We identified EUR, ICE and SVA as populations identical to those revealed by the PCA. Population FAR was shown to be an admixture of EUR and ICE (Fig. 2).

### Summary statistics

**Fig. 3:**
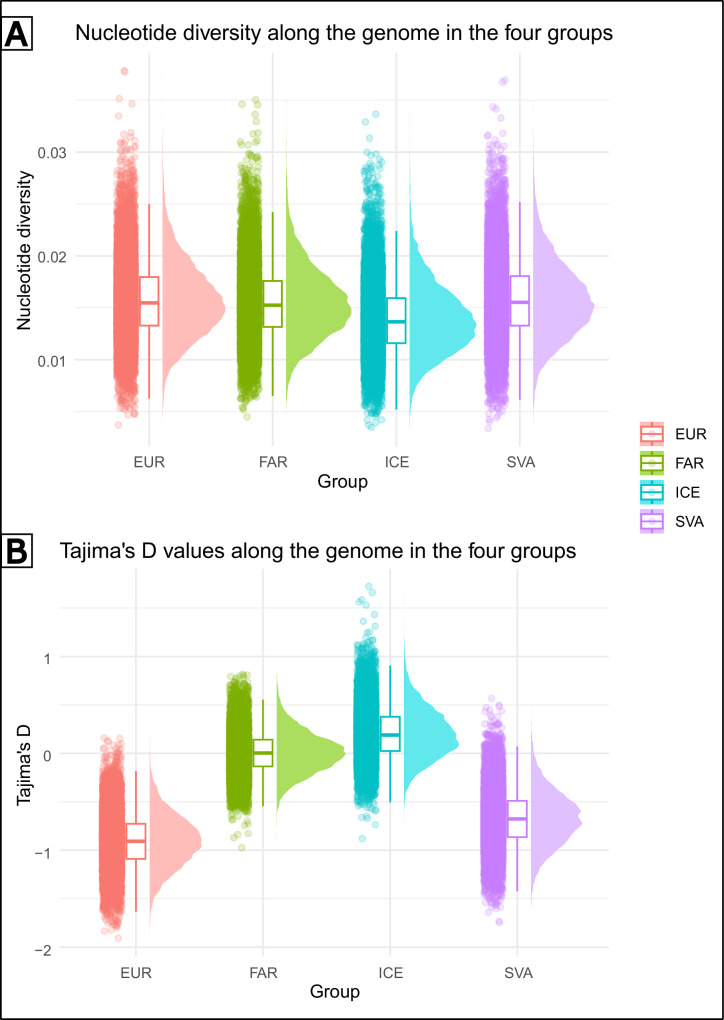
Genome-wide summary statistics. Raincloud plots of the genome-wide distribution of nucleotide diversity (**A**) and Tajima’s D (**B**) values within the four populations identified by the structure analyses.

Nucleotide diversity values for the four studied populations had similar values, with only those of Iceland being slightly lower (Fig. 3A). Tajima’s *D* values averaged close to 0 within FAR and ICE populations, while EUR and SVA exhibited, on average, negative Tajima’s D along the genome (Fig. 3B). For average and mean Tajima’s D and nucleotide diversity values, see Table 1.

**Table 1:** Average and median values of Tajima’s D and Nucleotide diversity in the four populations identified by the structure analyses.

Population	EUR		FAR		ICE		SVA	
	average	median	average	median	average	median	average	median
Tajima’s D	−0.9062	−0.9074	0.0091	0.0042	0.2054	0.1889	−0.6734	−0.6773
Nucleotide diversity	0.0157	0.0155	0.0155	0.0153	0.0139	0.0136	0.01578	0.0155

### Demographic inference

**Fig. 4:**
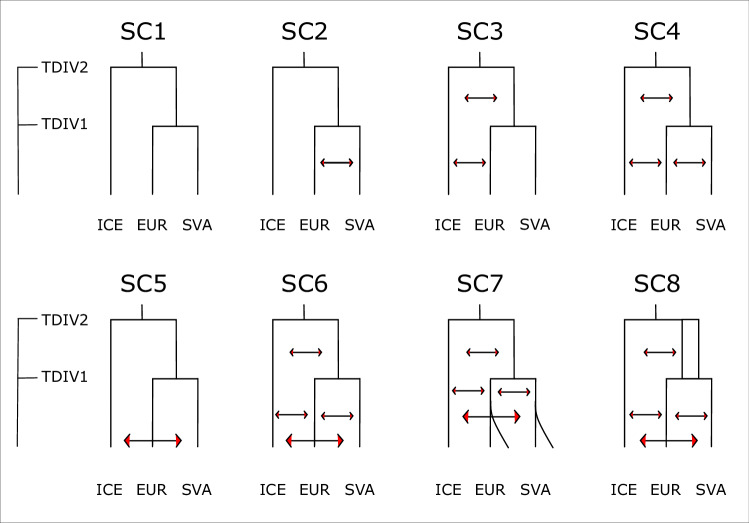
Diagrams showing the eight demographic scenarios which were tested in fastsimcoal. SC1–SC6 were designed to test the likelihood of the exchange of genes between the different populations. The following two scenarios then tested for the presence of population growth in the SVA and EUR populations. Exponential population growth following the EUR-SVA divergence was implemented in SC7, while SC8 accounted for population expansion, which predates the aforementioned split.

Out of the eight tested scenarios (Fig. 4) the maximum likelihood algorithm calculated the lowest likelihood difference between expected and observed data for scenario SC8. This scenario allowed for asymmetrical migration between all three pairs of populations. Additionally, an increased population size was implemented for the ancestral population of Europe and Svalbard. According to the parameters inferred from SC8 the divergence between EUR and SVA happened ca. 7468 generations ago (for *S. balanoides* we treat one generation as 1 year, https://www.marlin.ac.uk/; Nunez et al. 2018), while the ancestral population of EUR and SVA diverged from ICE ca. 21,011 ya (for all inferred parameters see Supplementary Table 3).

## Discussion

The Atlantification of the Arctic has been a hot topic in polar science. There have been several studies discussing the effects of the associated processes on living organisms, particularly, on their inhabited ranges. (Poloczanska et al. 2013; Hastings et al. 2020; Melo-Merino et al. 2020). In this study, we have attempted for the first time to discover the timing behind the wide, present-day distribution of the northern acorn barnacle *Semibalanus balanoides* in Svalbard. We have focused our attention on two alternative hypotheses that could help to explain this boreal species’ origin beyond the Arctic Circle.

More than 100,000 rigorously quality filtered and independent (i.e. LD-pruned) SNPs have helped us to unveil the presence of a particularly strong, geography influenced population structure of the species in the studied region. Our population structure analyses revealed four divergent populations: Scandinavia and British Isles (EUR), Faroe Islands (FAR), Iceland (ICE) and lastly Spitsbergen and Bjornoya Island (SVA). EUR and SVA seem to be closely related to each other, while ICE is more divergent, in fact, more closely related to the sole Western Atlantic sample in our dataset (Supplementary Fig. 1). Both the PCA and the admixture analysis suggest that the FAR population of *S. balanoides* is admixed between EUR and ICE (Figs. 1 and 2).

This strong genetic structure is a surprising finding, given that *S. balanoides* is considered highly dispersive due to the presence of pelagic larval stage in its life cycle (Anderson 1994). Another widely distributed barnacle, *Balanus improvisus* for example, lacked population structure in European waters (Wrange et al. 2016). Similarly, a recent single, mitochondrial marker-based study suggested the presence of a panmictic *S. balanoides* population in the North Atlantic (Csapó et al. 2023). Our results suggest that populations from different geographic regions do not seem to form such a panmictic population. Another study by Nunez et al. (2018) also reported a similar, distinct population structure between Eastern and Western Atlantic *S. balanoides* populations, based on pool-sequencing whole genome data. All this points out that in the case of marine species with large distribution areas, single markers might not provide sufficient information to infer population structure and demography.

One underlying reason for the population structure revealed between geographic regions might be old subdivisions of those populations followed by reduced gene flow. The results of the population structure analyses alone would suggest the existence of such divergent populations, which have potentially separated long ago, allowing for enough time to accumulate this level of differentiation.

An alternative explanation for the observed phenomenon could be the consideration of a more recent divergence between populations followed by enhanced genetic drift (e.g. after a strong bottleneck event) (Adkison 1995; Funk et al. 2016). Such an event could eliminate the shared polymorphism between populations and further increase the divergence.

In order to gain a better understanding of historical demography we examined two important population indices, namely nucleotide diversity and Tajima’s D within each of the four populations. While average and median nucleotide diversity values did not significantly differ between populations, Tajima’s D statistics were negative in EUR and SVA. From the point of view of demographic history these results may hint at population expansion within these areas (Schmidt and Pool 2002). At the same time, genome wide nucleotide diversity and Tajima’s *D* values calculated within each population did not show signs of a strong bottleneck including in SVA, which goes against the alternative explanation behind the examined population structure.

According to the conducted demographic reconstruction, ICE diverged from the ancestor of EUR and SVA ca. ~20 kya. This inferred date coincides with the end of the LGM in the northern hemisphere (Hughes et al. 2013). On the onset of glacial retreat, formerly uninhabitable coastlines became available for marine organisms, which had resided in refugia. The distribution range of *S. balanoides* along the eastern Atlantic reaches to the Iberian Peninsula on the south (Herrera et al. 2019). Iberean coasts as well as the British Isles and the English Channel could have served as a refugium for *S. balanoides* as they did for many other marine species during the LGM (Hoarau et al. 2007; Olsen et al. 2010). The sole sample from Rhode Island was more genetically related to ICE suggesting the Western Atlantic is probably not the source of Svalbard colonisation.

In accordance with our conclusions based on the population structure and summary statistics, results of the demographic reconstruction suggested that Arctic (Svalbard and Bjornoya) populations diverged from European ones ca. 7.5 kya. This result contradicts the hypothesis according to which boreal *S. balanoides* colonised Svalbard as a result of contemporary Atlantification processes. Instead, our results suggest this Arctic colonisation occurred during an earlier warmer cycle. Although the retreat of the Fenno-Scandinavian ice sheet which covered major parts of northern Europe and the north-eastern Atlantic area started ca. 22–17 kya, subsequent colder periods (e.g. the Younger Dryas between ca 13 and 11 kya) could have delayed or interrupted the recolonisation of coastlines beyond the Arctic circle. A major warm event known as the Holocene Thermal Optimum (HTO) at ca. 11–5 kya has been reported to significantly shape the faunal and floral composition of both marine and terrestrial postglacial ecosystems globally (Renssen et al. 2009; Salonen et al. 2011; Osika et al. 2022). It has been noted that post glacial warming was not homogenous, but rather provided several opportunities for boreal fauna and flora to claim roles in Arctic ecosystems (Mangerud and Svendsen 2018; Csapó et al. 2023). For example the blue mussel *Mytilus edulis* has been shown to colonise Svalbard after the end of the LGM, but then during a colder period disappeared from the archipelago (Berge et al. 2005). The species then recolonised Svalbard in recent years possibly as a result of enhanced Atlantic advection and human aid (Leopold et al. 2018; Kotwicki et al. 2021). We could not reveal a similar history for *S. balanoides*, even though the two species have largely overlapping distribution ranges, live in the same type of habitats and have highly dispersive larvae.

Postglacial warming and the subsequent shifts in species distribution are in many cases coupled with population expansion (Hewitt 2004). Following the detection of negative Tajima’s *D* values in EUR and SVA, we have implemented population growth for both of these populations in the demographic reconstruction. Although the model with recent growth proved to be less likely, an alternative explanation arose when increased ancestral population size was accounted for (SC8). Unfortunately, when it comes to the implementation of ancestral population growth, the programme fastsimcoal is limited, thus we could not acquire a date estimate for the actual start of the growth. Based on our hypothesis it is likely that population expansion started during the HTO and was followed by a split around 7.5 kya as shown by the results. This way, the genetic signal of this demographic event remained strong enough to be detected in both present-day daughter populations.

One remaining factor we need to account for is the potential effect of contemporary Atlantification on the gene flow between EUR and SVA populations. There is a clearly identified heterogeneity in the genetic make-up of the SVA population along the second axis (PC2) of the PCA plot (Fig. 1B), showing a gradient in ancestry towards EUR. Such a signal could point to the recent upsurge of gene flow between these two populations, a process that could be fuelled by present day Atlantification. The increased (Atlantic Water) AW inflow to Svalbard potentially transporting larvae from Scandinavia could contribute to this genetic Atlantification. The species have been described to encrust on moving/floating substrata (e.g. ships, plastic debris), which could be an alternative vector of long-distance dispersal (Van Sebille et al. 2016). Some individuals of the species were already found on the surface of macroplastic washed up at the shores of Spitsbergen (Weslawski and Kotwicki 2018). At the same time, it is worth noting that *S. balanoides* is an r-strategist, and the recruitment of larvae onto hard substrata is facilitated by a high propagule pressure, i.e. mass abundance of larvae in the water column (Bertness et al. 1992). Thus the transfer of single individuals by floating debris might not be sufficient to maintain a high constant gene flow between distant regions. The finer substructuring of the SVA population (Supplementary Fig. 2) might suggest two separate colonisation events. However, it is hard to interpret such a signal given the sample size differences between locations. To pinpoint the exact location of refugial areas within the studied regions is not a straightforward task. One potential source area can be the Lofoten archipelago in Norway, where the West Spitsbergen sea current branches out from the Norwegian coastal current and could directly transfer larvae to Svalbard. In fact, the Andoya island, closely located to Lofoten served as a cryptic glacial refugium for both terrestrial (Parducci et al. 2012) and marine species (Coyer et al. 2011). *S. balanoides* could have survived the LGM in these areas, then colonised Svalbard during the HTO utilising the increased AW advection. Verifying this scenario, together with the potential of the presence of adaptive signal along the latitudinal gradient would require further sampling effort and data exploration.

## Conclusions

The results of this study have presented several pieces of evidence against recent Atlantification aiding the colonisation of the Svalbard archipelago by the barnacle *S. balanoides*. The distinct population structure of Svalbard individuals, the lack of genetic footprint of a recent bottleneck and the demographic reconstruction suggest that the divergence of the Svalbard population occurred much earlier than the Anthropocene, most likely during the HTO (ca. 10–8 kya). At the same time we do not exclude the potential of an ongoing ‘genetic Atlantification’ (gene flow from EUR to SVA), where due to present day processes (e.g. human-induced connectivity) an upsurge in gene flow from Europe to Svalbard introduces ancestry heterogeneity in the Arctic population.

### Benefit sharing

A research collaboration was developed with scientists from the countries providing genetic samples, all collaborators are included as co-authors.

## Supplementary information

Supplementary Figure 1

Supplementary Figure 2

Supplementary Table 1

Supplementary Table 2

Supplementary Table 3

## Data Availability

Raw sequence reads are deposited in the SRA (BioProject PRJNA1128561).
