# Supplementary material for: Genome sequence analysis provides evidence that a boreal crustacean colonised Svalbard well before the ongoing Atlantification of the Arctic
Source: Heredity (Edinb). 2025 Aug 23;134(9):558–66. doi: 10.1038/s41437-025-00793-7 (PMC12457588; doi:10.1038/s41437-025-00793-7)
Supplement: Supplementary file 1 — Supplementary Figure 1 [file 41437_2025_793_MOESM1_ESM.docx]

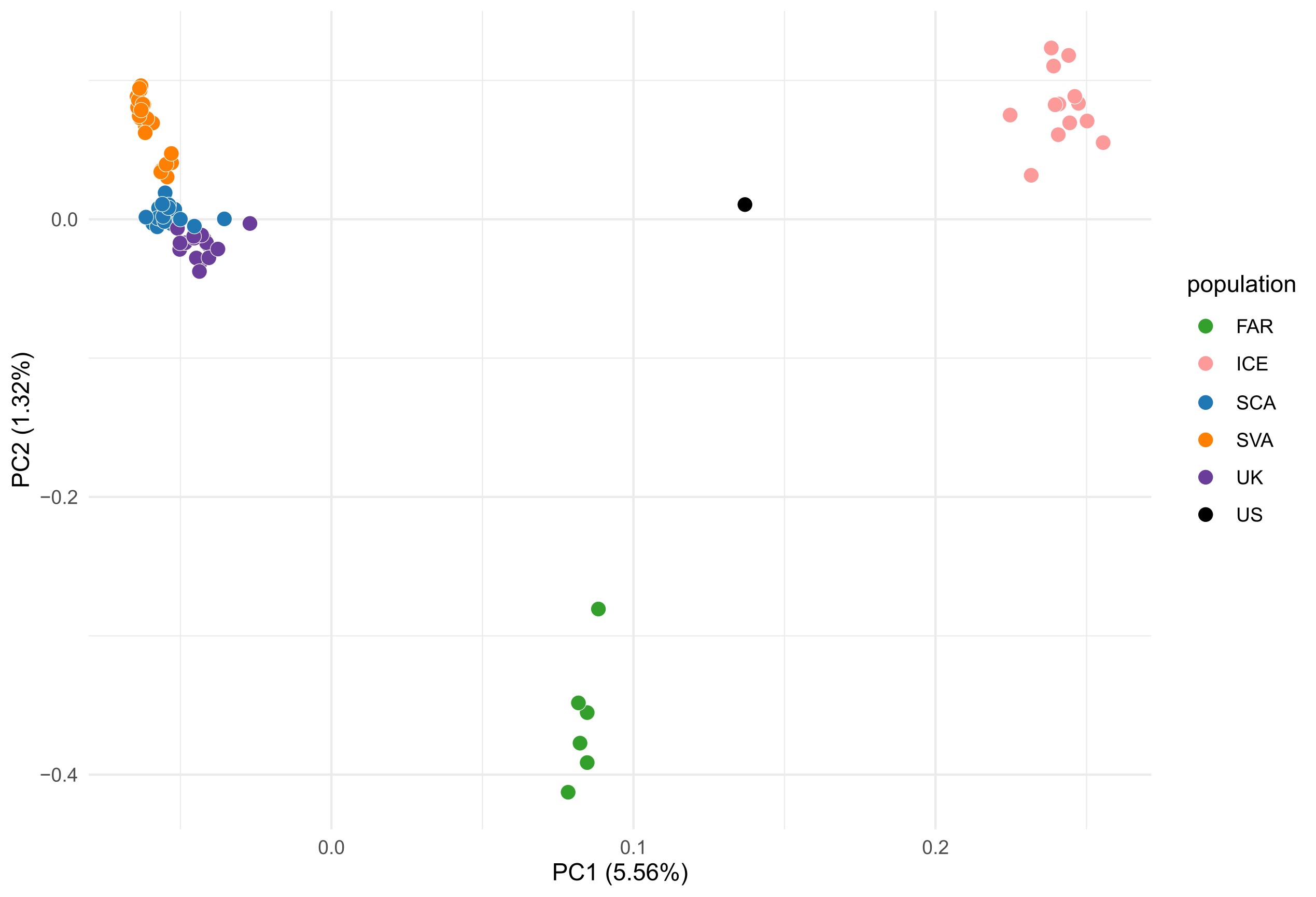


Supplementary figure 1. Principal component analysis (PCA) showing the structuring of genetic differences between all samples including one sample from the western Atlantic coast (Rhode Island).
