# Supplementary material for: Genome sequence analysis provides evidence that a boreal crustacean colonised Svalbard well before the ongoing Atlantification of the Arctic
Source: Heredity (Edinb). 2025 Aug 23;134(9):558–66. doi: 10.1038/s41437-025-00793-7 (PMC12457588; doi:10.1038/s41437-025-00793-7)
Supplement: Supplementary file 2 — Supplementary Figure 2 [file 41437_2025_793_MOESM2_ESM.docx]

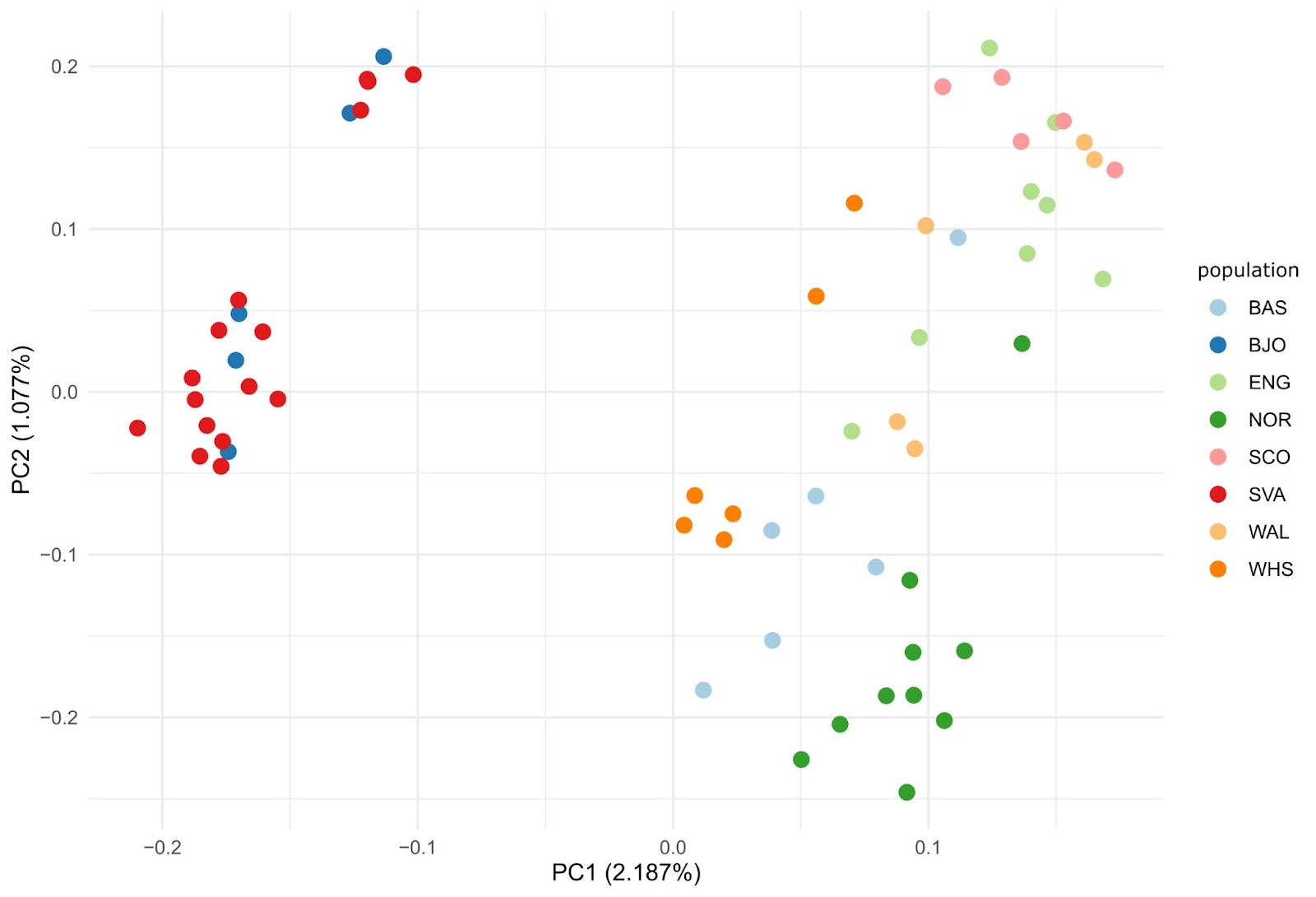


Supplementary figure 2. Principal component analysis (PCA) showing the structuring of genetic differences between samples from the EUR and SVA population. Barents Sea (BAR), Bjornoya Island (BJO), England (ENG), Norwegian Sea (NOR), Scotland (SCO), Svalbard (SVA), Wales (WAL), White Sea (WHS).
