# Supplementary material for: Genome sequence analysis provides evidence that a boreal crustacean colonised Svalbard well before the ongoing Atlantification of the Arctic
Source: Heredity (Edinb). 2025 Aug 23;134(9):558–66. doi: 10.1038/s41437-025-00793-7 (PMC12457588; doi:10.1038/s41437-025-00793-7)
Supplement: Supplementary file 3 — Supplementary Table 1 [file 41437_2025_793_MOESM3_ESM.docx]

Supplementary table 1. Sampling sites and assigned regions.

| Site name | Assigned region | Latitude | Longitude | Number of ind. |
| --- | --- | --- | --- | --- |
| BAS01 | Scandinavia | 69.0533 | 33.1997 | 2 |
| BAS03 | Scandinavia | 69.1011 | 36.0633 | 4 |
| BJO03 | Svalbard | 74.4658 | 19.2686 | 5 |
| ENG01 | United Kingdom | 55.4485 | -1.5862 | 5 |
| ENG02 | United Kingdom | 55.071 | -1.4499 | 2 |
| FAR01 | Faroes | 62.2166 | -7.0076 | 3 |
| FST4 | Faroes | 62.2509 | -7.0739 | 3 |
| ICE02 | Iceland | 63.8357 | -21.0652 | 9 |
| ICE09 | Iceland | 66.0939 | -17.3148 | 3 |
| ISF03 | Svalbard | 78.5871 | 14.5267 | 5 |
| KRO01 | Svalbard | 79.1497 | 11.8436 | 6 |
| NO05 | Scandinavia | 70.0245 | 21.286 | 5 |
| NO07 | Scandinavia | 69.6591 | 18.8265 | 4 |
| SCO02 | United Kingdom | 58.6468 | -3.3431 | 5 |
| SVA03 | Svalbard | 77.96 | 18.4744 | 5 |
| WAL01 | United Kingdom | 51.7871 | -5.1057 | 4 |
| WAL03 | United Kingdom | 51.7357 | -5.2471 | 1 |
| WHS01 | Scandinavia | 66.975 | 32.683 | 6 |
