# Supplementary material for: Genome sequence analysis provides evidence that a boreal crustacean colonised Svalbard well before the ongoing Atlantification of the Arctic
Source: Heredity (Edinb). 2025 Aug 23;134(9):558–66. doi: 10.1038/s41437-025-00793-7 (PMC12457588; doi:10.1038/s41437-025-00793-7)
Supplement: Supplementary file 4 — Supplementary Table 2 [file 41437_2025_793_MOESM4_ESM.docx]

Supplementary table 2. Calculated likelihoods for the admixture analyses from k = 1-7.

| K | 1 | 2 | 3 | 4 | 5 | 6 | 7 |
| --- | --- | --- | --- | --- | --- | --- | --- |
| Likelihood | Inf | Inf | 2649.55 | 1141.47 | 749.51 | 1158.5 | 1442.41 |
