# Supplementary material for: Genome sequence analysis provides evidence that a boreal crustacean colonised Svalbard well before the ongoing Atlantification of the Arctic
Source: Heredity (Edinb). 2025 Aug 23;134(9):558–66. doi: 10.1038/s41437-025-00793-7 (PMC12457588; doi:10.1038/s41437-025-00793-7)
Supplement: Supplementary file 5 — Supplementary Table 3 [file 41437_2025_793_MOESM5_ESM.docx]

Supplementary table 3. Parameter estimates from the most likely scenario (SC8) inferred by fastsimcoal. All parameters besides ANC2 (which served as a reference for optimisation) were corrected by the rescaling factor (r).

| **Name of the parameter** | **Description** | **Inferred value** |
| --- | --- | --- |
| NEUR | Effective population size of EUR | 43 181 |
| NICE | Effective population size of EUR | 1 389 622 |
| NSVA | Effective population size of EUR | 1 114 151 |
| ANC1 | EUR-SVA ancestral population size | 54 781 |
| ANC2 | EUR-ICE ancestral population size | 835 061 |
| TDIV1 | Divergence time between EUR-SVA | 7468 |
| TDIV2 | Divergence time between EUR-ICE | 21 011 |
| MUTRATE | Genome wide mutation rate of *S. balanoides* | 2.86e-09 |
| r | Rescaling factor | 14.5 |
| estimated likelihood =  -137396174.628 | observed likelihood =  -135026835.9 | likelihood difference =  2369338.68 |
